# The Application of Nano-TiO_2_ Photo Semiconductors in Agriculture

**DOI:** 10.1186/s11671-016-1721-1

**Published:** 2016-11-28

**Authors:** Yan Wang, Changjiao Sun, Xiang Zhao, Bo Cui, Zhanghua Zeng, Anqi Wang, Guoqiang Liu, Haixin Cui

**Affiliations:** 1 Institute of Environment and Sustainable Development in Agriculture, Chinese Academy of Agricultural Sciences, Beijing, People’s Republic of China; 2 Nanobiotechnology Research Center, Chinese Academy of Agricultural Sciences, Beijing, People’s Republic of China

**Keywords:** Nano-TiO_2_, Photo semiconductors, Agriculture, Pesticide

## Abstract

Nanometer-sized titanium dioxide (TiO_2_) is an environmentally friendly optical semiconductor material. It has wide application value in many fields due to its excellent structural, optical, and chemical properties. The photocatalytic process of nano-TiO_2_ converts light energy into electrical or chemical energy under mild conditions. In recent years, the study and application of nano-TiO_2_ in the agricultural sector has gradually attracted attention. The nano-TiO_2_ applications of degrading pesticides, plant germination and growth, crop disease control, water purification, pesticide residue detection, etc. are good prospects. This review describes all of these applications and the research status and development, including the underlying principles, features, comprehensive applications, functional modification, and potential future directions, for TiO_2_ in agriculture.

## Review

### Introduction and Background

**Fig. 1:**
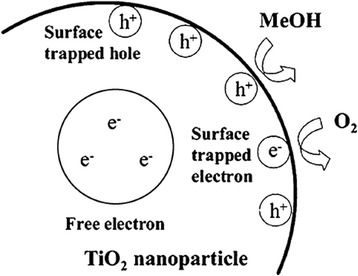
Spatial distribution of photogenerated charge carriers in TiO_2_ [16]

To date, the photo semiconductor, titanium dioxide (TiO_2_), has been proven to be the most effective and useful photocatalyst for both fundamental research and practical applications due to its high-efficiency, photochemical stability, nontoxic nature, and low cost [1–4]. Much research has been performed to explore the photocatalytic activity of TiO_2_ photo semiconductors in their nano form [5–7]. The photocatalytic activity mechanism of nano-TiO_2_ has been extensively studied in the literature [8–13], and the basic photocatalytic mechanism is shown in Fig. 1. Upon absorption of light energy larger than the band gap of TiO_2_, electrons are excited from the valence band to the conduction band, which creates electron (e^−^)–hole (h^+^) pairs. These charge carriers can rapidly migrate to the surfaces of catalyst particles, where they are ultimately trapped and undergo redox chemistry with suitable substrates. Thus, the trapped hole can react with chemisorbed OH^−^ or H_2_O to produce the OH· radicals. Oxygen, which is present in the system, acts as an efficient electron scavenger. Additionally, any other oxidant, such as OH^−^, can trap electrons [14–16].

Nano-TiO_2_ photo semiconductors have many applications in many fields including photocatalysis, agriculture, dye-sensitized solar cells, and biomedical devices [17]. However, in the agricultural field, the use of TiO_2_ nanomaterials is relatively new and requires further exploration. The nano-TiO_2_ photo semiconductor continues to attract attention of agricultural researchers because of its favorable physical/chemical properties, low cost, availability, and high stability. Thus, nano-TiO_2_ photo semiconductors have many application possibilities in agriculture including degradation of pesticides, plant protection, and residue detection. However, one disadvantage of TiO_2_ nanomaterials is that they are mostly active in the presence of UV light due to their large band gap of approximately 3.2 eV [18, 19]. The UV regime is only a small fraction of the Sun’s energy (<10%) [20]. Therefore, this property limits the application of TiO_2_ nanomaterials in agriculture, and the highly efficient use of TiO_2_ nanomaterials is sometimes prevented. Thus, several approaches have been developed to alleviate this problem and to improve the photocatalytic activity of TiO_2_ nanomaterials for a wide range of applications. One effective method for improving the performance of TiO_2_ nanomaterials is to increase their optical activity by shifting the response onset from the UV to the visible region by doping the TiO_2_ nanomaterial with different metals or other elements [21].

This paper aims to review and summarize the recent applications and research on nano-TiO_2_ photo semiconductors and their doping complexes in agriculture. The topics include pesticide degradation, plant germination and growth, crop disease control, water purification, and pesticide residue detection.

### Application of TiO_2_ Photo Semiconductors in Agriculture

#### Pesticide Degradation

Pesticides are widely used in agriculture, although their excessive usage may create hazards to both humans and the environment. Repeated use of pesticides results in a frequent occurrence of residues in the environment and in biota. Most pesticide residues require effective treatment and further removal due to their toxicity, high chemical stability, and low biodegradability. Consequently, considerable efforts have been devoted to developing methods that can remove residual pesticides and destroy bio-recalcitrant organic contaminants [22]. Semiconductor photocatalysis is a promising approach to remedy the pesticide residue problem, and it has attracted significant attention [14, 23]. TiO_2_ is the most investigated photo semiconductor. For the past decade, it has been widely studied as an efficient photocatalyst for pesticides [14].

**Fig. 2:**
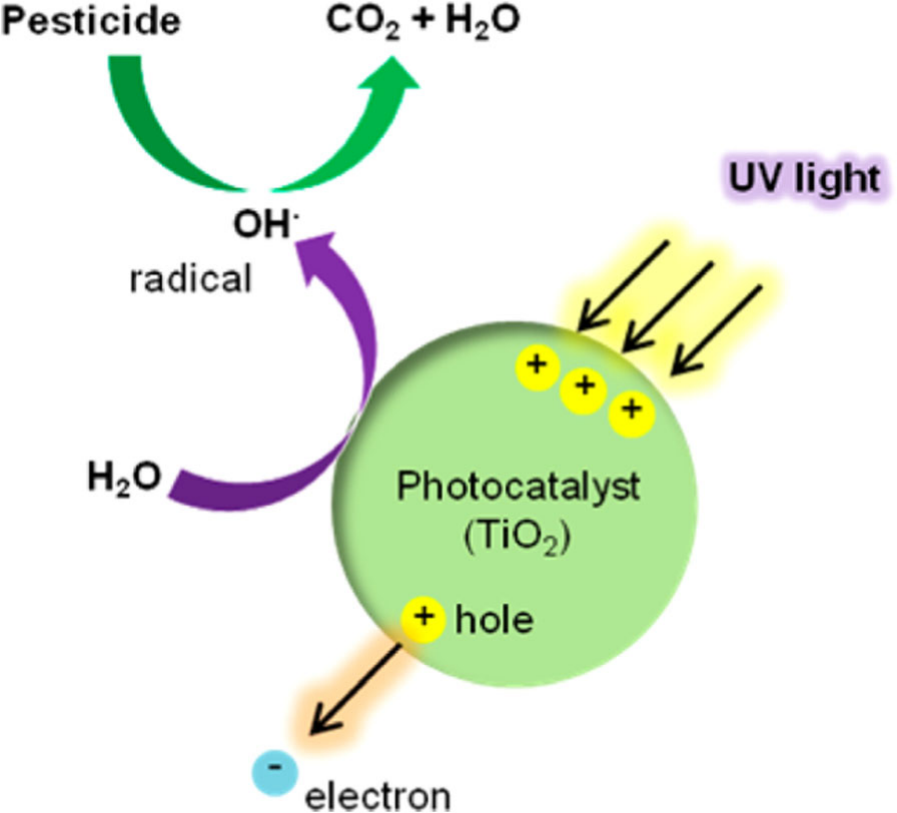
Schematic of the pesticide degradation mechanism: photocatalytic oxidation of TiO_2_ [24]

**Fig. 3:**
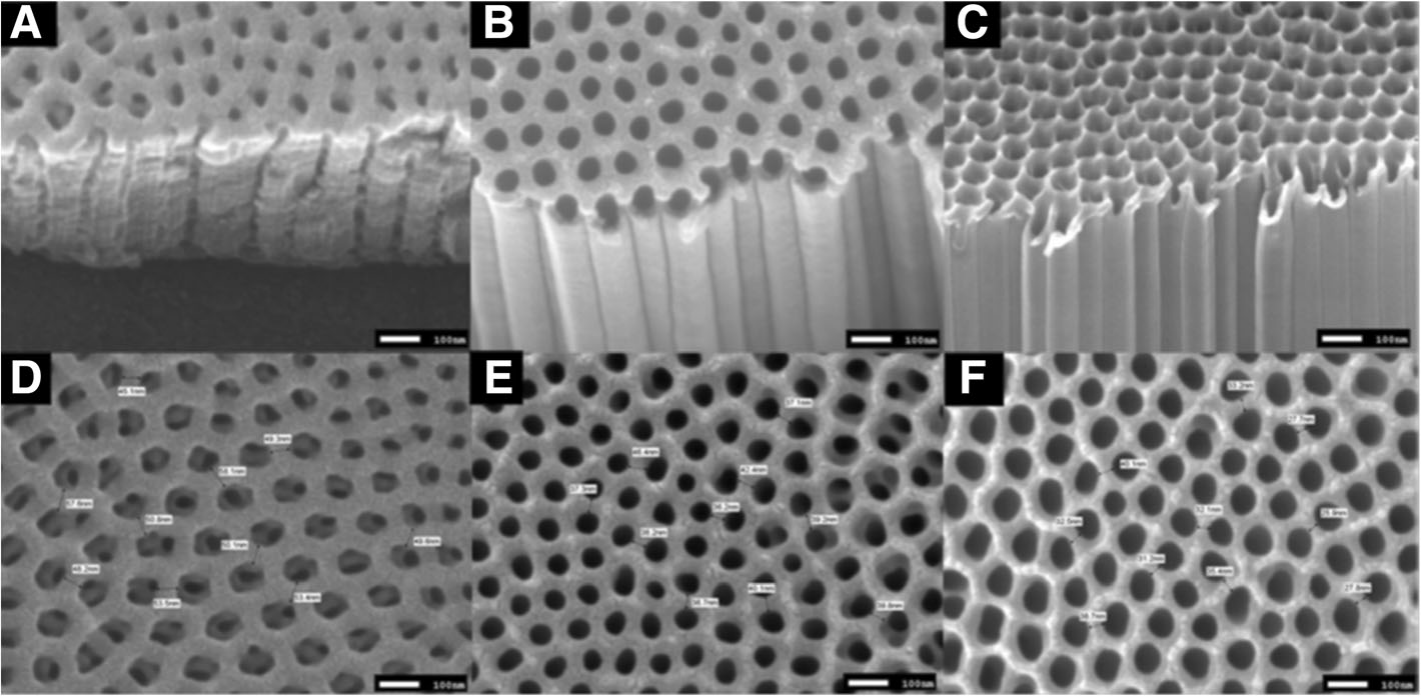
SEM analysis—side and top view for various tube lengths: 1.5 μm (**a**, **d**), 7 μm (**b**, **e**), and 10 μm (**c**, **f**) [30]

In the photocatalytic oxidation process, pesticides are destroyed in the presence of TiO_2_ photocatalysts and a UV light source. As illustrated in Fig. 2, when TiO_2_ is irradiated with photons whose energy is equal to or greater than its band gap energy (Eg = 3.2 eV), electron–hole pairs are created. In an aqueous system, these holes react with H_2_O or OH^−^ that are adsorbed on the semiconductor surface to produce OH· radicals, which are the strongest oxidants in this process [24–27]. These radicals react with pesticides that are adsorbed on the surface and decompose the pesticides [20]. The pesticides are degraded into H_2_O, CO_2_, and other biologically degradable and less toxic substances without secondary pollution. Rabindranathan reported that the TiO_2_ photocatalyst is effective in degrading the phosphamidon insecticide [28]. Several factors (e.g., phosphamidon concentration, pH of the system, catalyst loading, and the presence of anions) influence the degradation rate [28]. Lhomme reported the photocatalytic degradation of chlortoluron and cyproconazole pesticides on TiO_2_-coated media, and the process was found to be effective in degrading and mineralizing the pesticides [29]. Recent studies have found that the TiO_2_ morphology plays a key role on the photocatalytic activity. As shown in Fig. 3, TiO_2_ nanotube length has significant effect on the photocatalytic degradation of paraquat [30], and short tubes with a small internal diameter exhibit poor photocatalytic activity because pollutant diffusion is ineffective. The result shows that the optimal activity was found for 7-μm-long TiO_2_ tubes, and tubes longer than 7 μm have thinner walls; thus, light is absorbed on a longer distance and pollutant has to diffuse further to reach the oxidizing species [30].

**Fig. 4:**
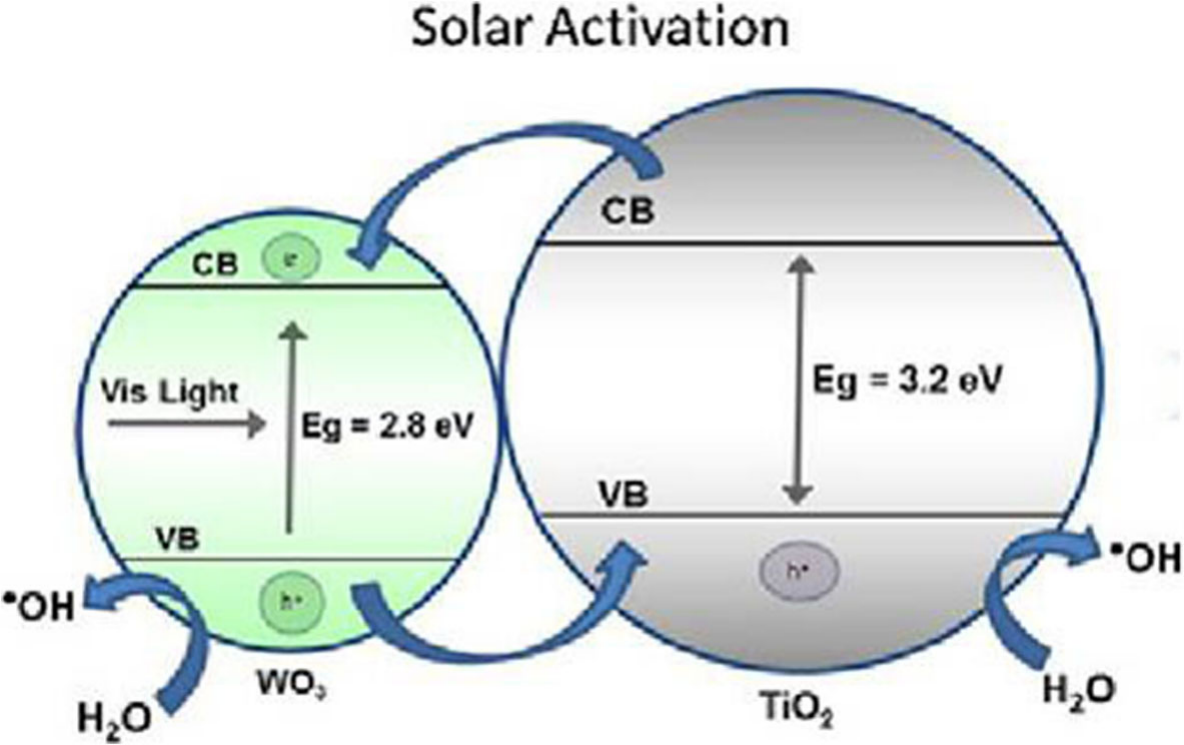
Solar photocatalytic activity of TiO_2_ modified with WO_3_ for the organophosphorus pesticide degradation [34]

This TiO_2_ photocatalytic degradation process of pesticides mainly relies on the in situ generation of highly reactive OH· radicals, which are capable of converting pesticides into relatively innocuous end products. However, the limitations for the wide application of TiO_2_ semiconductors for pesticide photocatalytic degradation include the high rate of electron–hole recombination, wide band gap, and inefficient visible light harvesting catalysts [31, 32]. Minimization of the electron–hole recombination and efficient visible light excitation are the major issues that increase the photocatalytic efficiency for pesticide degradation. To overcome these problems, many modifications have been applied to TiO_2_ nanomaterials such as doping with a metal coating, surface sensitization, surface area increase or designing, and developing secondary mixed oxides [33, 34]. The modifications of TiO_2_ nanomaterials with different special modifiers, such as Ag^+^, WO_3_, and W, have enhanced the pesticide photocatalytic degradation properties of the catalyst [35, 36]. An efficient charge separation can be obtained by coupling two semiconductor particles that have different energy levels [34, 37]. Figure 4 presents WO_3_ doping as an example. WO_3_ (Eg = 2.8 eV) can function as an electron accepting species in the presence of visible light, which is favorable for producing electron–hole pairs and for improving the pesticide photocatalytic degradation [34].

A substantial amount of research has been focused on modifying the TiO_2_ photocatalyst for enhancing pesticide degradation [22]. Ramos-Delgado et al. reported the solar photocatalytic activity of TiO_2_, which was modified with WO_3_, for degrading the organophosphorus pesticide [34]. The TiO_2_ semiconductor, which was loaded with 2% WO_3_, exhibited better solar photocatalytic behavior for degrading the malathion pesticide compared with bare TiO_2_. This was attributed to the formation of smaller clusters and a higher surface area, which reduced the electron–hole recombination process and resulted in a better contact area between the catalyst particles and the pesticide. Thus, the photocatalytic reactivity and efficiency were improved [34]. Guan et al. reported a W/TiO_2_ catalyst that was constructed for the photocatalytic degradation and mineralization of avermectin insecticide microcapsules. The catalyst had the highest photocatalytic activity with a 4.0 mol% W-doped amount due to the presence of electron-trapping centers (W^6+^) in W-doped TiO_2_ solid solutions. Guan et al. reported different types of TiO_2_-based photodegradable nano-imidacloprid insecticides [14]. Photocatalysts, including TiO_2_, sodium dodecyl sulfate (SDS)/TiO_2_, Ag/TiO_2_, and SDS/Ag/TiO_2_, were constructed for the photocatalytic degradation of the nano-imidacloprid insecticide. The photocatalytic activity of SDS/Ag/TiO_2_ was the highest among all of the photocatalysts due to its large specific surface area compared with TiO_2_, which led to the fast adsorption of reactants and enrichment of the insecticide. Moreover, depositing silver in the SDS/Ag/TiO_2_ photocatalyst significantly promotes the photocatalytic activity.

#### Plant Germination and Growth

**Fig. 5:**
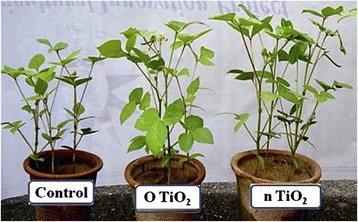
Phenology of a mung bean plant under various treatments (control, O TiO_2_: ordinary titanium dioxide, n TiO_2_: nano titanium dioxide) [42]

In recent years, various researchers have studied the effects of nanomaterials on plant germination and growth to promote the use of nanomaterials for agricultural applications [38]. TiO_2_ nanomaterials can induce active oxygen, including superoxide and hydroxide anions, in the photocatalytic process, which increases the seed stress resistance and water and oxygen intake. These are required for the fast germination of plants. Zheng et al. reported the effects of TiO_2_ photocatalysts on the growth of spinach seeds. They demonstrated that the nano-TiO_2_-treated seeds that were produced from plants that had a higher dry weight, higher photosynthetic rate, and increased chlorophyll formation. This suggested that TiO_2_ nanomaterials promoted the absorption of inorganic nutrients and increased the photosynthetic rate [39]. The research results of Song et al. showed that the effect on plant growth was more pronounced with TiO_2_ nanoparticles than with bulk TiO_2_. TiO_2_ nanoparticles stimulated plant growth at low concentrations but inhibited plant growth at high concentrations [40]. Yang showed that spinach leaves could be kept green using nano-anatase TiO_2_ treatment due to N_2_ fixation. Additionally, the fresh weight, dry weight, and the content of total nitrogen, NH_4_^+^, chlorophyll, and protein in spinach were clearly increased [41]. As shown in Fig. 5, Raliya et al. reported the physiological effects of TiO_2_ nanoparticles in mung bean. The results demonstrated a significant increase in plant growth for plants that were treated with TiO_2_ nanoparticles. In the control, plants exposed to TiO_2_ nanoparticles showed significant improvements in shoot length, root length, root area, and root nodules [42].

### Crop Disease Control

Conventional bactericidal methods that are used to protect plants against pathogens apply chemical pesticides to the irrigation water. However, this method of controlling plant diseases is hazardous both to humans and to the environment. Photochemical disinfection of plant pathogens using TiO_2_ thin films offers an alternative method for preventing plant pathogens [43]. The TiO_2_ photocatalyst technique has a potential for agricultural applications because it does not form dangerous compounds [44]. Under light, TiO_2_ nanomaterials generate superoxide ion radicals and hydroxides. These active oxygen species are effective antimicrobial agents. In recent years, various researchers have studied the effect of nano-TiO_2_ photo semiconductors in controlling crop diseases. However, UV accounts for only approximately 3% of the solar light spectrum. This limits the TiO_2_ photocatalytic disinfection application under visible light irradiation [44]. Yao et al. reported that the TiO_2_ thin film photocatalytic efficiency is improved under visible light by doping with a novel photosensitive dye. Thus, phytopathogenic bacteria in vegetable crops can be effectively inhibited by visible light irradiation. Cui et al. studied the bactericidal effect of nano-TiO_2_ on cucumbers [45]. The nano-TiO_2_ formed a successive, adhesive, and transparent film on the surface of the leaves. Further, the nano-TiO_2_-treated cucumber leaves had powerful bactericidal effects on plant pathogens due to the photocatalytic and photo biological effects of TiO_2_, which inhibited bacterial and fungal diseases.

#### Water Purification

In recent years, the growing concern about the problem of water decontamination from organic pollutants during agricultural production has led to research on methods that improve the efficiency and lower the consumption of chemical reagents [46]. Because photocatalysts use solar energy, the photocatalytic decomposition of organic pollutants in water is of particular interest and has received significant attention from scientists [47–50]. TiO_2_ is the most popular semiconductor that is used in photocatalytic processes [47–50]. TiO_2_ photo semiconductors that are a large size are stoichiometric and thus exhibit poor photocatalytic activity. However, nano-TiO_2_ crystallites (typical size <50 nm) have the expected electronic properties for applications in photocatalysis because of their higher activity [51]. In this photocatalysis process, reactive species can be formed on the surface of a nano-TiO_2_ photocatalyst that is exposed to UV radiation. The complete degradation and mineralization of a large variety of organic contaminants can be achieved in most cases [52, 53].

However, the main problem, which has limited the practical application of nano-TiO_2_ photocatalysis for water purification, is either a relatively low process rate or a limited efficiency for the use of irradiated energy [1, 54, 55]. A possible approach to solve this problem is the exploitation of low-cost radiation such as solar energy [56, 57]. However, the intensity of ultraviolet radiation in the solar spectrum is very limited. Therefore, the use of metals or metal oxide doping to extend the TiO_2_ absorption to the visible range is currently a good option for solving the problem. This approach enhances the photocatalytic activity of TiO_2_ and improves the utilization efficiency of the radiation energy [20, 58]. For instance, it was determined that the addition of fluoride to TiO_2_ significantly enhances the degradation rate of phenol [22, 59]. The dominant parameters (e.g., dopant nature, dopant concentration, and thermal treatment) affect the material [60]. Vione reported that fluoride addition to TiO_2_ enhanced the photocatalytic degradation of many organic compounds that were transforming via different pathways [46]. Bessekhouad reported that alkaline-doped TiO_2_ at low concentrations could be a promising material to degrade organic pollutants. The best results were obtained for 5% Li-doped TiO_2_ that was prepared using the impregnation technique [51]. Brezová et al. reported that the presence of metals, such as Li^+^, Zn^2+^, Cd^2+^, Pt^0^, Ce^3+^, Mn^2+^, Al^3+^, and Fe^3+^, could significantly change the photoactivity of TiO_2_ that was prepared using the sol–gel technique [60]. In addition, the effect of doping TiO_2_ with Li and Rb was studied by López et al., and the obtained materials were used to decompose 2,4-dinitroaniline [59].

#### Pesticide Residue Detection

Depending on their aqueous solubility, pesticides either remain in the soil or enter surface waters and ground waters. Pesticide degradation residues can remain in vegetables, animals, and water sources and can become more concentrated as they move up the food chain. There is an increasing interest in developing systems to sense, monitor, and remove pesticide residues because they are toxic even at trace levels.

Currently, pesticide detection methods typically use liquid or gas chromatography coupled with mass spectrometric detection (HPLC-MS and GC-MS) due to the sensitivity and reliability of these techniques. However, these approaches require meticulous sample preparation and highly qualified technicians [61]. Nanomaterial-based sensors can be used to detect pesticide residues. These nanosensors are alternatives to traditional methods due to their high sensitivity, low detection limits, high selectivity, fast response, and small size. Because of their simplicity, low cost, and ease of miniaturization, electrochemical and optical biosensors are widely used for detecting pesticides.

During recent decades, nano-TiO_2_ photo semiconductors, which are efficient sorbents for enriching and detecting pesticides, have attracted significant attention in the photocatalytic and photoelectrochemical area due to their nontoxicity, hydrophilicity, availability, and stability against photocorrosion. Additionally, they have a suitable flat band potential and are easily supported on various substrates [62–65].

TiO_2_ was used as an efficient and selective sorbent to recognize the phosphorylation moiety based on a strong chelation with phospho-moieties. The affinity of TiO_2_ towards the phosphoric group is favorable for fast enrichment and detection of free organophosphate pesticides [19]. However, the wide band gap of TiO_2_ (∼3.2 eV, anatase) allows it to absorb only the ultraviolet light (<387 nm). To extend its photo response to the visible region and to promote the photoelectric conversion efficiency, many modification methods have been applied (e.g., dye sensitization, metal ion/nonmetal atom doping, semiconductor coupling, and noble metal deposition) [19, 66]. Of the abovementioned methods, by considering the high electron mobility of nanocrystals and the possibility of shifting the optical band gap to the visible light region using organic materials, the organic–inorganic heterojunction can produce a robust photoelectrochemical sensor. Zhou reported that graphene-modified TiO_2_ nanotube arrays exhibit an excellent enrichment efficiency for carbamate pesticides including metolcarb, carbaryl, isoprocarb, and diethofencarb. The detection limits of these carbamate pesticides range from 2.27 to 3.26 μg L^−1^. The method could be used as a faster and easier alternative procedure for routine analysis of carbamate pesticides [67]. Li et al. developed two photoelectrochemical sensors to detect dichlofenthion and chlorpyrifos pesticides. The sensors were based on a TiO_2_ photocatalyst coupled with electrochemical detection, which is a derivative of an electrochemical sensor and sensitized TiO_2_ [68, 69].

## Conclusions

Over the past decades, nano-TiO_2_ has shown its potential for agricultural applications because of its high photocatalytic disinfection and photo biological effects coupled with its low price, nontoxicity, and stable performance. The continuous breakthroughs in the synthesis and modifications of TiO_2_ nanomaterials have resulted in new properties and new agricultural applications including pesticide degradation, plant germination and growth, crop disease control, water purification, and pesticide residue detection with improved performance. The research demonstrates that nano-TiO_2_ photo semiconductors are essential for degrading organic pollutants, preventing and controlling plant diseases with an antiviral or antibacterial function, and protecting the environment. These characteristics provide new approaches for solving environmental pollution and pesticide residue problems in agriculture.
